# Seasonal Fluctuations in Atopic Dermatitis: A Global Perspective Using Google Trends Data

**DOI:** 10.1177/12034754241265713

**Published:** 2024-07-26

**Authors:** Mahan Maazi, Nima Nasiri, Shanti Mehta, Eric P. McMullen, Muskaan Sachdeva, Jensen Yeung

**Affiliations:** 1 Faculty of Medicine, University of British Columbia, Vancouver, BC, Canada; 2 Department of Electrical and Computer Engineering, University of British Columbia, Vancouver, BC, Canada; 3 Temerty Faculty of Medicine, University of Toronto, Toronto, ON, Canada; 4 Michael G. DeGroote School of Medicine, McMaster University, Hamilton, ON, Canada; 5 Division of Dermatology, Department of Medicine, Women’s College Hospital, Toronto, ON, Canada

**Keywords:** atopic dermatitis, eczema, Google Trends, development index

To the Editor:

Despite the increasing recognition of seasonal factors in atopic dermatitis (AD) research, there remains an absence of comprehensive, large-scale investigations studying seasonal trends and AD. Previous literature has reported a seasonal pattern of AD, peaking in the spring, summer, and winter.^1^ One study from South Korea found that AD symptoms in children were the most severe in spring, winter, and autumn compared to summer.^2^ However, these prior analyses are limited to individual countries. To our knowledge, this is the first analysis on seasonality and AD severity at a population level using Google Trends (GT) data.

Patients will use Google when faced with new symptoms, diagnosis, or treatment. Due to this, GT has become a popular tool to understand search behavior. In dermatology, GT has been used to look at patterns, social media trends, and seasonality of certain conditions.^3^ Using worldwide data can be inaccurate for seasonality trends because countries in northern and southern hemispheres are combined, and data are influenced by a country’s access to the internet, mobile device or computer, and Google search engine. One way of addressing this is by using the widely accepted Information and Communication Technology Sector Development Index (IDI).^4^ The IDI is a publicly accessible measurement tool used to assess the universal use and connectivity of internet access in participating countries (169 in 2023).^4^

Our study used a time series analysis with Kruskal-Wallis and Dunn’s tests with Bonferroni correction to determine significant differences in GT search interest across months from 2004 to January 2024 for search term “Atopic eczema” and “Atopic dermatitis” averaged in 42 countries (Supplemental File 1). Countries were included if they had an IDI score of near or over 72.8 (world average) and high enough search volume index (SVI; above 10) to adjust for outliers or were added if they were not included in the IDI but had a high SVI. We did not conduct a study on the southern hemisphere as 9 countries were represented in the final cutoff, leading to high variability. Our analysis revealed seasonality exists within data (H-statistic = 206.07, *P* value = 4.07e-38), and Dunn test with Bonferroni correction shows that differences in SVI are statistically significant between January and May compared with July to October and December (Supplemental Files 2 and 3).

Results indicate that Google searches for AD peaks from January to May compared with July to October and December in our 42 countries. This aligns with previous data and studies. Although our results represent multiple countries with differing climate and temperatures, physicians should be aware of this seasonality. Increased searches may indicate additional inquiry, support, and management for patients. Limitations of this study include only using northern hemisphere data, search trend factors unrelated to flares (AD in the news, campaigns, etc), the use of only AD and atopic eczema as a (averaged across countries) search term, and patients using other search engines or resources beyond Google. Future research should investigate additional countries including the southern hemisphere, other possible confounding factors such as temperature and humidity, and including additional search terms.

## Supplemental Material

sj-docx-1-cms-10.1177_12034754241265713 – Supplemental material for Seasonal Fluctuations in Atopic Dermatitis: A Global Perspective Using Google Trends Data Supplemental material, sj-docx-1-cms-10.1177_12034754241265713 for Seasonal Fluctuations in Atopic Dermatitis: A Global Perspective Using Google Trends Data by Mahan Maazi, Nima Nasiri, Shanti Mehta, Eric P. McMullen, Muskaan Sachdeva and Jensen Yeung in Journal of Cutaneous Medicine and Surgery

sj-docx-2-cms-10.1177_12034754241265713 – Supplemental material for Seasonal Fluctuations in Atopic Dermatitis: A Global Perspective Using Google Trends Data Supplemental material, sj-docx-2-cms-10.1177_12034754241265713 for Seasonal Fluctuations in Atopic Dermatitis: A Global Perspective Using Google Trends Data by Mahan Maazi, Nima Nasiri, Shanti Mehta, Eric P. McMullen, Muskaan Sachdeva and Jensen Yeung in Journal of Cutaneous Medicine and Surgery

sj-docx-3-cms-10.1177_12034754241265713 – Supplemental material for Seasonal Fluctuations in Atopic Dermatitis: A Global Perspective Using Google Trends Data Supplemental material, sj-docx-3-cms-10.1177_12034754241265713 for Seasonal Fluctuations in Atopic Dermatitis: A Global Perspective Using Google Trends Data by Mahan Maazi, Nima Nasiri, Shanti Mehta, Eric P. McMullen, Muskaan Sachdeva and Jensen Yeung in Journal of Cutaneous Medicine and Surgery

sj-xlsx-4-cms-10.1177_12034754241265713 – Supplemental material for Seasonal Fluctuations in Atopic Dermatitis: A Global Perspective Using Google Trends Data Supplemental material, sj-xlsx-4-cms-10.1177_12034754241265713 for Seasonal Fluctuations in Atopic Dermatitis: A Global Perspective Using Google Trends Data by Mahan Maazi, Nima Nasiri, Shanti Mehta, Eric P. McMullen, Muskaan Sachdeva and Jensen Yeung in Journal of Cutaneous Medicine and Surgery This article is distributed under the terms of the Creative Commons Attribution 4.0 License ( http://www.creativecommons.org/licenses/by/4.0/) which permits any use, reproduction and distribution of the work without further permission provided the original work is attributed as specified on the SAGE and Open Access pages ( https://us.sagepub.com/en-us/nam/open-access-at-sage).
