# Supplementary material for: Seasonal Fluctuations in Atopic Dermatitis: A Global Perspective Using Google Trends Data
Source: J Cutan Med Surg. 2024 Jul 26;28(5):494–5. doi: 10.1177/12034754241265713 (PMC11523540; doi:10.1177/12034754241265713)
Supplement: sj-docx-1-cms-10.1177_12034754241265713 – Supplemental material for Seasonal Fluctuations in Atopic Dermatitis: A Global Perspective Using Google Trends Data [file sj-docx-1-cms-10.1177_12034754241265713.docx]

**Supplementary File 1**.

Countries' IDI score, included and excluded with reason. Red cells indicate countries removed either for being an outlier (low search volume index (SVI)) or Southern Hemisphere countries which were ultimately excluded due to high variability. Green cells indicate included countries (42 total). Blue cells indicate countries that were around or slightly below the IDI cutoff but were included due to their significant SVI. Yellow cells indicate countries that were not assessed at all in the IDI but were added due to their significant SVI.

| **Country** | **IDI Score (Out of 100)**  **World average (72.8)** | **Included?** | **Reason Not Includes** |
| --- | --- | --- | --- |
| Albania | 81.6 | No | Low SVI; outlier |
| Algeria | 77.8 | No | Low SVI; outlier |
| Andorra | 87.2 | No | Low SVI; outlier |
| Antigua and Barbuda | 79.7 | No | Low SVI; outlier |
| Argentina | 81.5 | No | Southern hemisphere; high variability |
| Armenia | 85.1 | No | Low SVI; outlier |
| Australia | 94.0 | No | Southern hemisphere; high variability |
| Austria | 92.5 | Yes | - |
| Azerbaijan | 79.0 | No | Low SVI; outlier |
| Bahamas | 88.5 | No | Low SVI; outlier |
| Bahrain | 96.5 | No | Low SVI; outlier |
| Barbados | 77.3 | No | Low SVI; outlier |
| Belarus | 86.9 | No | Low SVI; outlier |
| Belgium | 88.2 | Yes | - |
| Bhutan | 76.5 | No | Low SVI; outlier |
| Bosnia and Herzegovina | 76.6 | No | Low SVI; outlier |
| Botswana | 74.0 | No | Low SVI; outlier |
| Brazil | 81.9 | No | Southern hemisphere; high variability |
| Brunei Darussalam | 94.8 | No | Low SVI; outlier |
| Bulgaria | 85.6 | Yes | - |
| Canada | 87.2 | Yes | - |
| Chile | 90.7 | No | Southern hemisphere; high variability |
| China | 84.4 | Yes | - |
| Colombia | 71.9 (SVI score below cutoff, but included due to significance in searches) | Yes | - |
| Costa Rica | 83.9 | No | Low SVI; outlier |
| Croatia | 87.1 | Yes | - |
| Cyprus | 87.4 | No | Low SVI; outlier |
| Czech Republic | 86.1 | Yes | - |
| Denmark | 96.9 | Yes | - |
| Dominica | 76.9 | No | Low SVI; outlier |
| Dominican Rep. | 75.0 | No | Low SVI; outlier |
| Egypt | 75.8 | No | Low SVI; outlier |
| Estonia | 96.9 | No | Low SVI; outlier |
| Fiji | 73.2 | No | Low SVI; outlier |
| Finland | 96.7 | Yes | - |
| France | 89.4 | Yes | - |
| Gabon | 72.9 | No | Low SVI; outlier |
| Georgia | 85.1 | No | Low SVI; outlier |
| Germany | 87.3 | Yes | - |
| Greece | 83.7 | No | Low SVI; outlier |
| Grenada | 73.4 | No | Low SVI; outlier |
| Hong Kong, China | 96.5 | No | Low SVI; outlier |
| Hungary | 86.8 | No | Low SVI; outlier |
| Iceland | 94.8 | No | Low SVI; outlier |
| India | No IDI but included due to high SVI | Yes | - |
| Indonesia | 80.1 | No | Southern hemisphere; high variability |
| Iran (Islamic Republic of) | 80.9 | No | Low SVI; outlier |
| Ireland | 88.9 | Yes | - |
| Israel | 91.1 | Yes | - |
| Italy | 86.4 | Yes | - |
| Jamaica | 77.0 | No | Low SVI; outlier |
| Japan | 92.0 | Yes | - |
| Jordan | 78.5 | No | Low SVI; outlier |
| Kazakhstan | 88.9 | No | Low SVI; outlier |
| Korea (Rep. of) | 93.8 | Yes | - |
| Kuwait | 98.2 | No | Low SVI; outlier |
| Kyrgyzstan | 84.7 | No | Low SVI; outlier |
| Latvia | 93.8 | No | Low SVI; outlier |
| Lebanon | 76.1 | No | Low SVI; outlier |
| Libya | 79.4 | No | Low SVI; outlier |
| Liechtenstein | 91.9 | No | Low SVI; outlier |
| Lithuania | 92.4 | No | Low SVI; outlier |
| Luxembourg | 92.1 | No | Low SVI; outlier |
| Macao, China | 93.3 | No | Low SVI; outlier |
| Malaysia | 94.5 | Yes |  |
| Maldives | 79.0 | No | Low SVI; outlier |
| Malta | 87.0 | No | Low SVI; outlier |
| Mauritius | 81.7 | No | Low SVI; outlier |
| Mexico | 78.0 | Yes | - |
| Moldova | 77.1 | No | Low SVI; outlier |
| Mongolia | 85.9 | No | Low SVI; outlier |
| Montenegro | 83.9 | No | Low SVI; outlier |
| Morocco | 85.1 | No | Low SVI; outlier |
| Netherlands (Kingdom of the) | 93.5 | Yes | - |
| New Zealand | 89.5 | No | Southern hemisphere; high variability |
| North Macedonia | 79.6 | No | Low SVI; outlier |
| Norway | 90.9 | Yes | - |
| Oman | 90.5 | No | Low SVI; outlier |
| Panama | 74.8 | No | Low SVI; outlier |
| Paraguay | 71.7 (SVI score below cutoff, but included due to high SVI) | Yes | - |
| Peru | 73.4 | No | Southern hemisphere; high variability |
| Poland | 94.6 | Yes | - |
| Portugal | 85.6 | Yes | - |
| Puerto Rico | No IDI but included due to high SVI | Yes | - |
| Qatar | 97.3 | No | Low SVI; outlier |
| Romania | 87.0 | Yes | - |
| Russian Federation | 88.9 | Yes | - |
| Saint Kitts and Nevis | 82.3 | No | Low SVI; outlier |
| Saint Lucia | 73.3 | No | Low SVI; outlier |
| Saint Vincent and the Grenadines | 73.0 | No | Low SVI; outlier |
| Saudi Arabia | 94.9 | Yes | - |
| Serbia | 85.1 | No | Low SVI; outlier |
| Seychelles | 80.9 | No | Low SVI; outlier |
| Singapore | 97.4 | Yes | - |
| Slovakia | 87.1 | No | Low SVI; outlier |
| Slovenia | 88.4 | Yes | - |
| South Africa | 80.5 | No | Low SVI; outlier |
| Spain | 91.4 | Yes | - |
| Suriname | 76.8 | No | Low SVI; outlier |
| Sweden | 93.9 | Yes | - |
| Switzerland | 91.6 | Yes | - |
| Taiwan | No IDI but included due to high SVI | Yes | - |
| Thailand | 88.7 | Yes | - |
| Trinidad and Tobago | 76.6 | Yes | - |
| Tunisia | 75.4 | No | Low SVI; outlier |
| Türkiye | 85.8 | Yes | - |
| Ukraine | 80.8 | Yes | - |
| United Arab Emirates | 96.4 | Yes | - |
| United Kingdom | 92.8 | Yes | - |
| United States | 96.6 | Yes | - |
| Uruguay | 87.1 | No | Southern hemisphere; high variability |
| Uzbekistan | 81.7 | No | Low SVI; outlier |
| Viet Nam | 80.6 | No | Low SVI; outlier |
