# Supplementary material for: Seasonal Fluctuations in Atopic Dermatitis: A Global Perspective Using Google Trends Data
Source: J Cutan Med Surg. 2024 Jul 26;28(5):494–5. doi: 10.1177/12034754241265713 (PMC11523540; doi:10.1177/12034754241265713)

**Supplementary File 2.**

Seasonal patterns of Google Trends ‘atopic dermatitis' data from January 2004 to December 2023, highlighting the seasonal pattern across the years. Data are displayed in yearly panels, showcasing the changes in seasonal patterns for each year. The x-axis displays the months, while the y-axis shows the standardized search interest values.


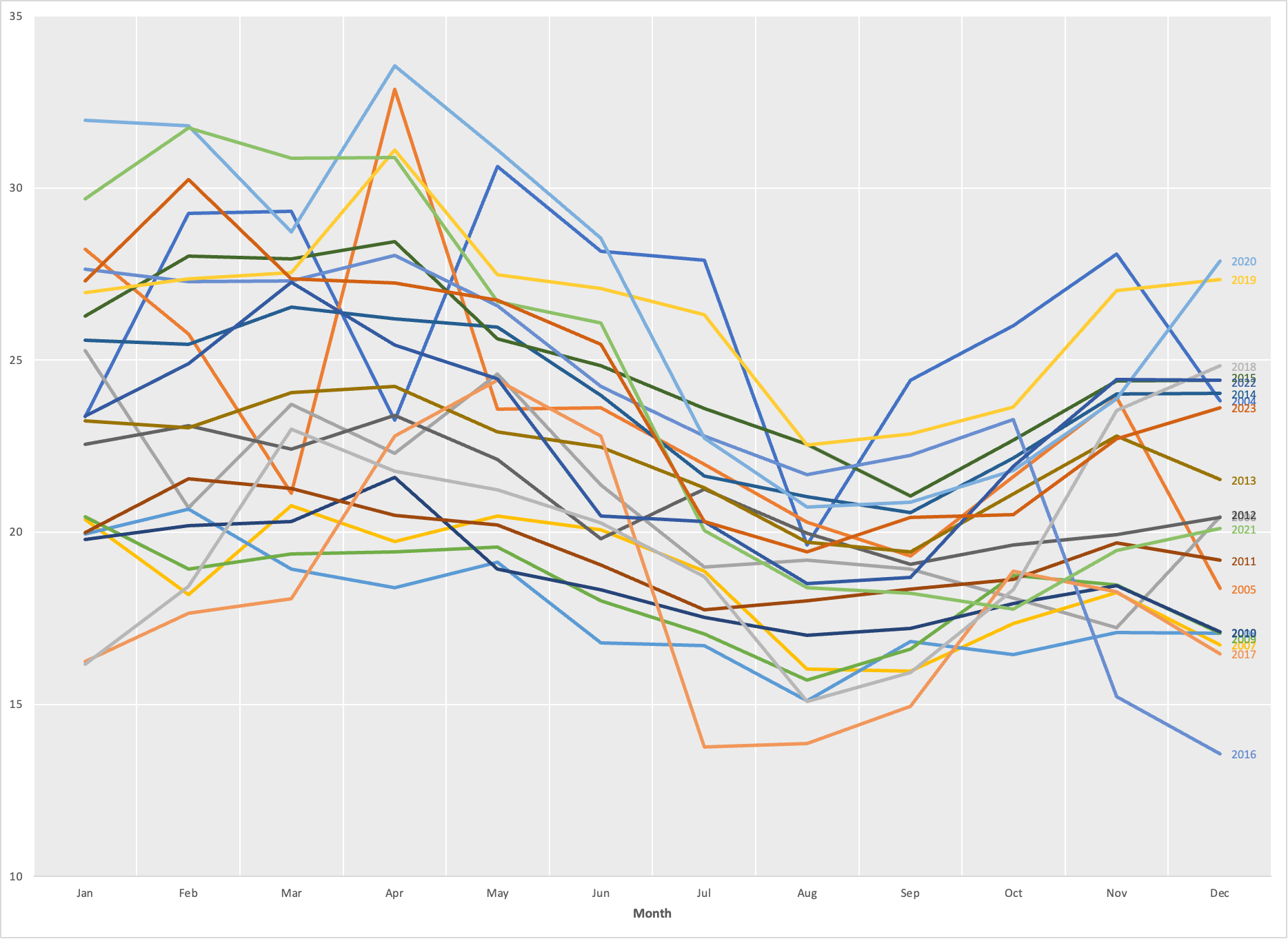

Supplement: sj-docx-2-cms-10.1177_12034754241265713 – Supplemental material for Seasonal Fluctuations in Atopic Dermatitis: A Global Perspective Using Google Trends Data [file sj-docx-2-cms-10.1177_12034754241265713.docx]
