# Supplementary material for: Seasonal Fluctuations in Atopic Dermatitis: A Global Perspective Using Google Trends Data
Source: J Cutan Med Surg. 2024 Jul 26;28(5):494–5. doi: 10.1177/12034754241265713 (PMC11523540; doi:10.1177/12034754241265713)
Supplement: sj-docx-3-cms-10.1177_12034754241265713 – Supplemental material for Seasonal Fluctuations in Atopic Dermatitis: A Global Perspective Using Google Trends Data [file sj-docx-3-cms-10.1177_12034754241265713.docx]

**Supplementary File 3.**

Average seasonal search interest by month—STL decomposition and minimal difference/first month to reach statistical significance by Dunn's test with Bonferroni correction.

| **Month** | **Average seasonal search interest** | **First month with statistical difference** | **Month interest difference** |
| --- | --- | --- | --- |
| Apr | 4.29 | Jun* | 3.93 |
| Mar | 3.46 | Nov* | 4.16 |
| Feb | 2.97 | Jun* | 2.61 |
| Jan | 2.74 | Jun* | 2.38 |
| May | 2.58 | Nov* | 3.28 |
| Jun | 0.36 | Jul* | 2.81 |
| November | -0.70 | - | - |
| December | -1.87 | - | - |
| July | -2.45 | - | - |
| October | -2.69 | - | - |
| August | -4.47 | - | - |
| September | -4.53 | - | - |

*Note*: p.adj: Represents the p-value calculated after Bonferroni correction.
